# Cell origin and genome profile difference of penoscrotum invasive extramammary Paget disease compared with its *in situ* counterpart

**DOI:** 10.3389/fonc.2022.972047

**Published:** 2022-08-24

**Authors:** Yamin Rao, Jinchao Zhu, Haiyan Zheng, Yong Ren, Tianhai Ji

**Affiliations:** ^1^ Department of Pathology, Ninth People’s Hospital, Shanghai Jiao Tong University School of Medicine, Shanghai, China; ^2^ Department of Pathology, General Hospital of Central Theater Command of the Chinese People’s Liberation Army (PLA), Wuhan, China

**Keywords:** cell origin, genome profiles, extramammary Paget disease (EMPD), invasive, *in situ*, penoscrotum, whole exome sequencing (WES), immunohistochemistry

## Abstract

Penoscrotum extramammary Paget disease (pEMPD) is a rare cutaneous carcinoma with an unknown cell origin. pEMPD always presents as a tumor *in situ* with an indolent process, whereas some progress into invasive forms with more aggressive behavior. The *in situ* and invasive cases display different morphologies and biological behavior, and thus far, a relationship between these two components has not been demonstrated. Immunohistochemistry was used to disclose the immunotype of pEMPD, and the results revealed that invasive/*in situ* pEMPD possessed with some identical immunophenotypes such as CK7, P63, and CK10, which inferred the clonal relatedness. The variable expressions of GCDFP-15 and carcino embryonic antigen hinted that tumor cell origin might be an epidermal sweat gland in epiderma. In our cohort, invasive pEMPD presented increased expression of androgen receptor and decreased MUC5CA expression, and these two changes might bring to the shift of invasive phenotype. To better understanding the relationship between these distinct tumor forms, we performed whole exome sequencing testing to evaluate overlapping genomic alterations of six paired invasive/*in situ* pEMPDs. The results showed that missense mutation was the predominant mutation type, and C>T transition accounted for 65.1% in all SNP mutation. Among the top 20 differential genes obtained from the six paired invasive/*in situ* pEMPD analysis, MUC4 (one missense, one in frame del, and one multi-hit), AHNAK2 (two missense and one multi-hit), DOT1L (two missense and one multi-hit), and FRG1 (two missense and one-multi hit) mutations were most enriched in invasive pEMPDs, which postulated that these genes may play roles in the disease progression.

## Introduction

Extramammary Paget disease (EMPD) is a rare skin malignancy with a slow clinical progression. Asian populations have higher prevalence of EMPD in men than in women. The most common anatomic site for men is penoscrotum, accounting for about 70% of EMPD cases (1). Primary EMPD is subdivided into forms according to whether tumor cells invade dermal stroma as *in situ*, in which tumor component was confined in epithelium and invasive (2). Most EMPD cases are diagnosed as carcinoma *in situ*, which is characterized by an insidious and indolent course with local recurrence rate ranging from 20% to 40% However, when the disease invades into the dermis and known as invasive EMPD, it possesses more aggressive features and has higher potential of regional lymph node metastases and distant metastases (3). Although the mutated genes of EMPD have been extensively studied (4–6), their roles in tumor progression have been scarcely addressed.

Until now, the cell origin of EMPD remains incompletely described. There are two main theories to explain the tumor cell origin. One is epidermotropic theory that suggests that tumor cells in the epidermis continuously come from underlying skin-associated glands adenocarcinoma cells (7). The other one is transformation theory that postulates that tumor cells originate *in situ* upon malignant transformation of basal keratinocyte, rather than from migrating adenocarcinoma cells (8). A better understanding of this could lead to improve models that are more reflective of the disease and further support preclinical therapeutic trial and preventive strategies needed to minimize the risk of developing EMPD.

Given that genetic alteration between *in situ* and invasive pEMPD and the cell origin of the disease was not clear, we planned to compare the oncogenic activities in invasive and *in situ* pEMPD samples to detect comprehensive molecular genetic profiles and immunophenotype of the disease from patients recruited from our center. Our primary objective was to identify previously unreported differential mutations between invasive and *in situ* groups by whole exome sequencing (WES) testing, which might be associated with promoting disease progression and/or be clinically actionable. Our secondary objective was to determine the cell origin of this specific area of EMPD through a panel of biomarkers performed by immunohistochemistry (IHC), given that the EMPD has a higher incidence in men in Asian countries.

## Materials and methods

### Data collection

Primary pEMPD cases (*in situ* and invasive) between January 2015 and December 2021 undergoing surgical resection at our hospital were retrospectively analyzed. All data came from the hospital electronic medicine record and pathology reporting system. All cases confirmed that no underlying malignancy was found so that it ruled out the possibility of secondary EMPD. Samples with insufficient quantity for subsequent immunohistological and molecular profiling tests were excluded. Finally, 153 patients were enrolled in this study. The histologic diagnosis and accompanying diagnostic immunohistochemical workup were reconfirmed in all cases. Our study was approved by the Human Ethics Committee of the Shanghai Nineth People’s Hospital, Shanghai Jiaotong University School of Medicine (SH9H‐2019‐T181‐2).

### Immunohistochemistry

Sections (4 mm thick) from formalin-fixed paraffin-embedded (FFPE) specimens from all 153 cases were prepared for IHC using a Dako automatic staining system (Autostainer Link 48). The primary antibodies including cytokeratin 7 (CK7), cytokeratin 20 (CK20), and c-erbB-2 [human epidermal growth factor receptor type 2 (HER-2)] were purchased from Dako. Gross cystic disease fluid protein (GCDFP-15), androgen receptor (AR), carcino embryonic antigen (CEA), P63, and mucin 5 subtype AC (MUC5AC) were from Gene Tech Inc. All steps were carried out according to the manufacturer’s protocol. A tumor cell was considered positive when cytoplasm (CK7, CK20, GCDFP-15, and CEA) or nuclear (P63) was stained. Immunostaining was noted in > 5% of tumor cells. AR nuclear staining in a fraction of neoplastic cells ≥ 1% was considered positive. Her-2 was evaluated according to the ASCO-CAP guidelines described for breast cancer (9); briefly, it was considered positive if >10% of cancer cells showed complete and circumferential (3+) expression or weak to moderate complete membrane staining observed in > 10% of tumor cells (2+).

### DNA extraction

Ten patients with pathologically confirmed pEMPD between 2018 and 2021 were considered for inclusion. Tumor specimens were recovered immediately after surgical resection. During the procedure, the surgeon uses a 4- to 6-mm punch biopsy knife to restore the tumor site. After trimming excess tissue to enrich for tumor cells, DNA was extracted from FFPE samples after microdissection using the GeneRead FFPE kit (Qiagen) according to the manufacturer’s protocol. Through the quality control test of 10 pairs of samples for the WES testing, the quality and quantity of DNA in four samples (two *in situ* and two invasive components, respectively) were insufficient for subsequent experiments, so we finally had six pairs of invasive/*in situ* samples for the next WES testing.

### Sequencing for WES testing

DNA libraries were prepared using SureSelect XT (Agilent), and the libraries were subjected to hybrid capture using SureSelect Human All Exon V6 (Agilent) according to the manufacturer’s protocol. Randomly breaking genomic DNA into a length of 180–280 bp by Covaris breaker after end-repairing and adding A-tail, the two ends of the fragment are connected with adapters to prepare DNA library liquid-phase hybridization with up to 500,000 biotin-labeled probes after pooling of the library with a specific index. Then, magnetic beads with streptomycin are used to capture n exons of n genes, and then PCR linear amplification is used to capture them. After the addition, the library quality inspection is carried out, and the sequencing can be carried out if it is qualified. Tumor purity in WES samples was assessed using Sequenza software. After library construction, the Qubit 2.0 was used for initial quantification, followed by the Agilent 2100 for initial quantification. The insert size of the library was detected. After the insert size was in line with expectations, the qPCR method was used to determine the size of the library. Effective concentration (3 nM) was considered for accurate quantification to ensure library quality. The library inspection is qualified, and the Illumina platform is used for the library according to the effective concentration and data output requirements of the library. Sequencing depth is 300× for both invasive/*in situ* parts.

### Mutation analysis of pEMPD

The “maftools” package in R software preliminarily analyzed and visualized the variation data for the cohort. “plotmafSummary” package was used to display the mutation type profile and then select the high mutant genes. To display the mutation information in the MAF file, “oncoplot” function visualizes differential mutated genes. titv function was used to classify Single Nucleotide Polymorphism (SNPs) into transitions and conversions, and bar graphs and stacked bar graphs were used to visualize the conversion forms between different bases.

### Compare mutation load against TCGA cohorts

The “tcgaCompare” was used to compare the mutation load of EMPD with the 33 cancer types in The Cancer Genome Atlas (TCGA). The “maftools” package was used the “tcgaCompare” function to provide the tumor mutation load in the MAF file and compare it with the 33 cancer types in TCGA.

### Coexistence or mutual exclusion analysis of differential mutant genes

To explore whether the mutant genes jointly affected the progress of EMPD, we used the “somaticInteractions” function to perform the pair-wise Fisher’s exact test on differential genes. The significance of co-mutation was different from that of single-gene mutation that would provide help for subsequent treatment and management for the disease.

### Statistical analysis

SPSS version 20 software (IBM Japan, Tokyo, Japan) was used for the statistical analyses. Continuous variables such as age are presented as means and standard deviations (mean ± SD), and categorical variables are presented as counts and percentages. We analyzed the differences between groups using the Pearson’s chi-square test or the Fisher’s exact test for categorical variables. All bioinformatics statistical analyses were also performed by R version 4.0.0. Two-sided P-values of <0.05 were regarded as statistically significant, and the significant statistical differences were defined as *, P <0.05; **, P <0.01; and ***, P <0.001.

## Results

### Clinical and pathological features of the enrolled patients

**Figure 1:**
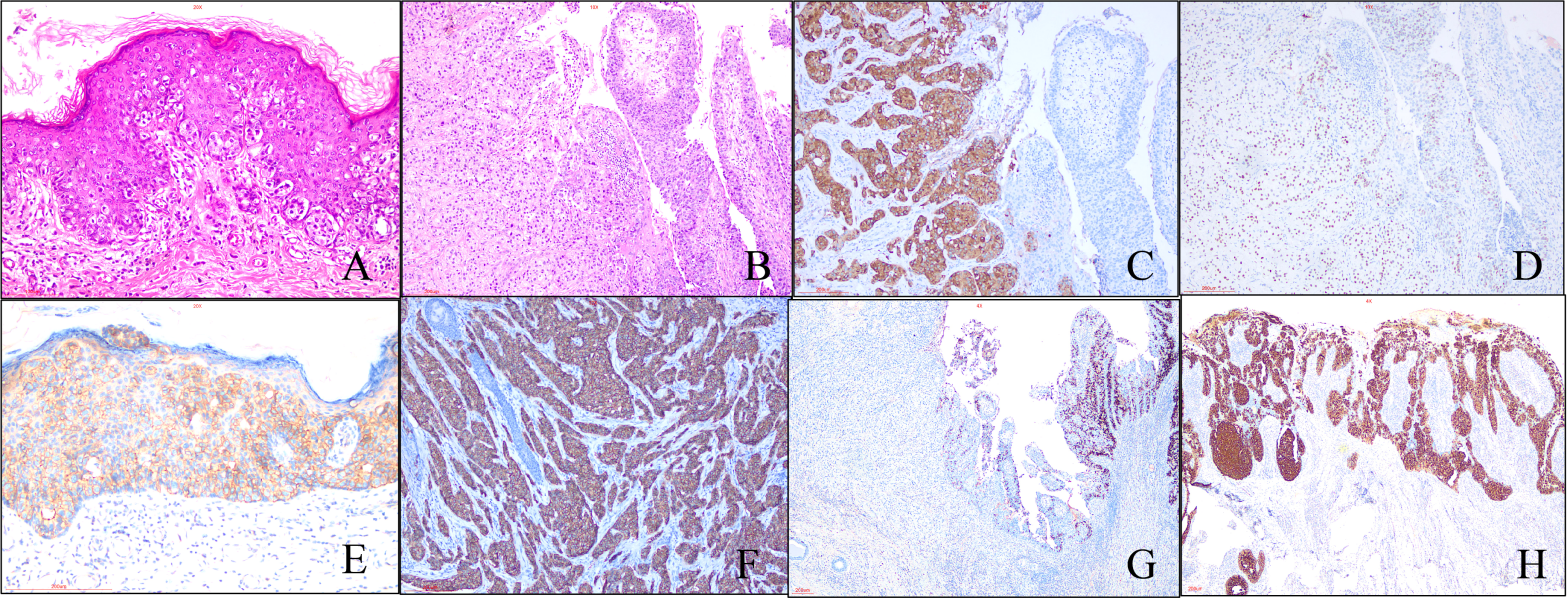
Morphological and immunohistochemical features of pEMPD. Hematoxylin and eosin staining reveals PEMPD in suit, in which PCs were located in intraepithelium and always involve the skin appendage. Characteristic intraepithelial PCs with a large and pale-staining vacuolated cytoplasm and prominent nuclei are present within the epidermis, showing a cranial spread. No invasion of the basement membrane is present. **(A)** (×200 magnification), and PCs can invade epidermal forming invasive pEMPD **(B)** (×100 magnification). **(C)** pEMPD can express GCDFP-15 (×100 magnification). **(D)** AR nuclear positivity (×100 magnification). Her-2 is evaluated as 2+ **(E)** (×200 magnification) and 3+ **(F)** (×100 magnification) according to the staining intensity and the number of positive tumor cells. **(G)** Cytoplastic MUC5CA expression is decreased or lost in invasive part of EMPD (×40 magnification). **(H)** Rarely, majority of PCs show CK20-positive (×40 magnification).

A total of 153 cases were enrolled in our study. Histologically, characteristic EMPD tumor cell is Paget cell (PC) that is an atypical large cell with abundant, clear, and sometimes eosinophilic cytoplasm. For *in situ* cases, PCs are arranged singly or in small groups, sometimes with the glandular formation in the basal layer of the epidermis especially located in lower part of the epidermis (**Figure 1A**) . Sometimes, PCs may spread into the contiguous epithelium of adnexal (hair follicle, eccrine gland, or sebaceous gland). Uncommonly, PCs may invade into the dermis and/or subcutaneous tissue with adenocarcinoma morphology (**Figure 1B**), forming invasive pEMPD. According to the degree of infiltration, EMPD can be divided the into three groups: *in situ* (confined to the epidermis), microinvasive (stromal invasion depth of ≤1 mm), and invasive (invasion depth of >1 mm) (10). We subclassified our cohort into two groups: *in situ* group (G1) (n = 72) and invasive group (G2) (n = 46) regardless of tumor invasive depth. Although the form of *in situ* was the predominant, the potential presence of stromal invasion was still a characteristic of pEMPD that was present in 30.1% of patients. It generally developed after the seventh decade of life and the mean age at onset was 71.0 ± 8.2 years (range, 51 to 89 years). For G1, the mean age at onset was 70.5 ± 8.658 years (range, 51 to 89 years). For G2, the mean age was 72.39 ± 6.987 years (range, 60 to 86 years).

### Immunohistochemistry

The results of IHC staining for 153 cases were summarized in **Table 1**. All cases were positive for CK7 and negative for P63, CK10, and CEA, which was expressed in majority cases (83.7%). Positive GCDFP-15 (**Figure 1C**) presented in more than half species, and positive percentage for G1 and G2 was close. We found the expression of AR in 69.3% of patients, 66.4% and 76.1% for G1 and G2, respectively (**Figure 1D**); there was no statistical significance between G1 and G2 (p = 0.2320). Overall, 47.1% of patients had a Her-2–positive tumor (**Figures 1E, F**), 43.9% and 54.3% in G1 and G2, respectively. pEMPD with MUC5CA-positive was 77.1%; the expression might decrease or loss (**Figure 1G**) in the invasive region with positive rates of 88.8% and 50.0% for G1 and G2, respectively. Variable CK20 was positive in eight of the 153 (5.2%) patients (**Figure 1H**): five of 107 (4.7%) patients in G1 and three of 46 (6.5%) patients for G2 with no significant difference. Statistical analysis results showed that only MUC5CA expression was associated with tumor invasion (p < 0.05).

**Table 1:** Evaluation of immunohistochemical markers in EMPDs.

Antibody	Subgroups	n	Positive (n)	Percent (%)	Negative (n)	Percent (%)	χ2	*P*-value
CK7	Total	153	153	100.0	0	0.0		
	G1	107	107	100.0	0	0.0		
	G2	46	46	100.0	0	0.0		
P63	Total	153	0	0.0	153	100.0		
	G1	107	0	0.0	107	100.0		
	G2	46	0	0.0	46	100.0		
CK10	Total	153	0	0.0	153	100.0		
	G1	107	0	0.0	107	100.0		
	G2	46	0	0.0	46	100.0		
CEA	Total	153	128	83.7	25	16.3	0.5010	0.4790
	G1	107	91	85.0	16	15.0		
	G2	46	37	80.4	9	19.6		
GCDFP-15	Total	153	81	52.9	72	47.1	0.2280	0.6330
	G1	107	58	54.2	49	45.8		
	G2	46	23	50.0	23	50.0		
AR	Total	153	106	69.3	46	30.7	1.4320	0.2320
	G1	107	71	66.4	36	33.6		
	G2	46	35	76.1	11	23.9		
Her-2	Total	153	72	47.1	81	52.9	1.4030	0.2360
	G1	107	47	43.9	60	56.1		
	G2	46	25	54.3	21	46.6		
MUC5CA	Total	153	118	77.1	35	22.9	27.4290	0 ***
	G1	107	95	88.8	12	11.2		
	G2	46	23	50.0	23	50.0		
CK20	Total	153	8	5.2	145	70.9	0.2220	0.6380
	G1	107	5	4.7	102	95.3		
	G2	46	3	6.5	43	93.5		

The significant statistical differences were defined as *, P <0.05; **, P <0.01; and ***, P <0.001.

### Comparison of gene mutations between invasive pEMPD and *in situ* pEMPD

**Figure 2:**
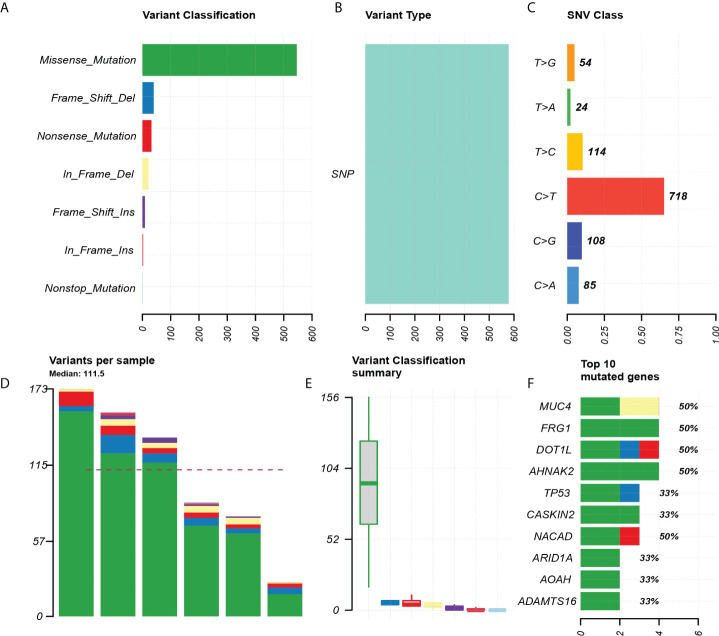
Exon mutation profile for six paired pEMPD samples. **(A)** Mutation classification, based on missense_ Mainly mutation. **(B)** SNP mutation type. **(C)** SNP mutation classification. **(D, E)** box plot for mutation types in each sample. **(F)** Top 10 differential genes.

Through the quality inspection of 10 pairs of samples for WES, the quality and quantity of DNA in four samples (two *in situ* and two invasive components, respectively) were insufficient for subsequent experiments, so we finally had WES data from six pairs of samples. The analysis showed that missense mutation (**Figures 2A, B**). SNP classification showed that C>T transition was predominant, accounting for 65.1%, to a lesser extent, T>C transition and C>G transversion (**Figure 2C**). The number of somatic alterations ranged from 26.30 to 173.30 mutations per exome with median of 111.5 mutations (**Figures 2D, E**). The top 10 mutated genes, including AHNAK2, DOT1L, FRG1, MUC4, and NACAD, were listed (**Figure 2F**).

**Figure 3:**
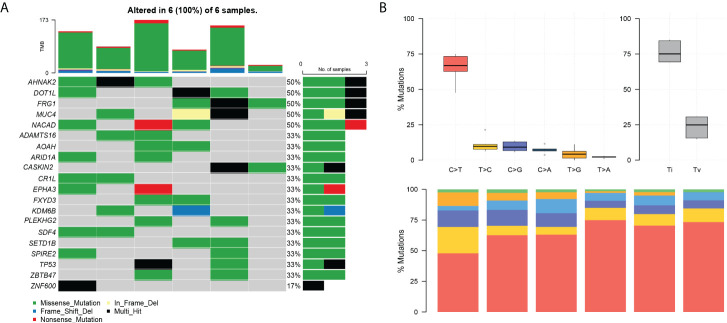
The landscape of differential mutations in paired pEMPD. **(A)** The Oncoprint of differential mutations shows that AHNAK2, DOT1L, FRG1, MUC4, and NACAD account for 50% of all mutant samples. The upper stacked bar plots illustrate the TMB in each sample. **(B)** The bar plots show the overall distribution of six SNP mutations; the stacked bar graph shows the transformation proportion in each sample.

Among the top 20 positively selected genes in the paired samples, AHNAK2, DOT1L, FRG1, MUC4, and NACAD were top five in the list, in which each presented in half cases (**Figure 3A**). C>T was the predominant mutation type showed by the conversion proportion in each sample (**Figure 3B**).

### Tumor mutation load

**Figure 4:**
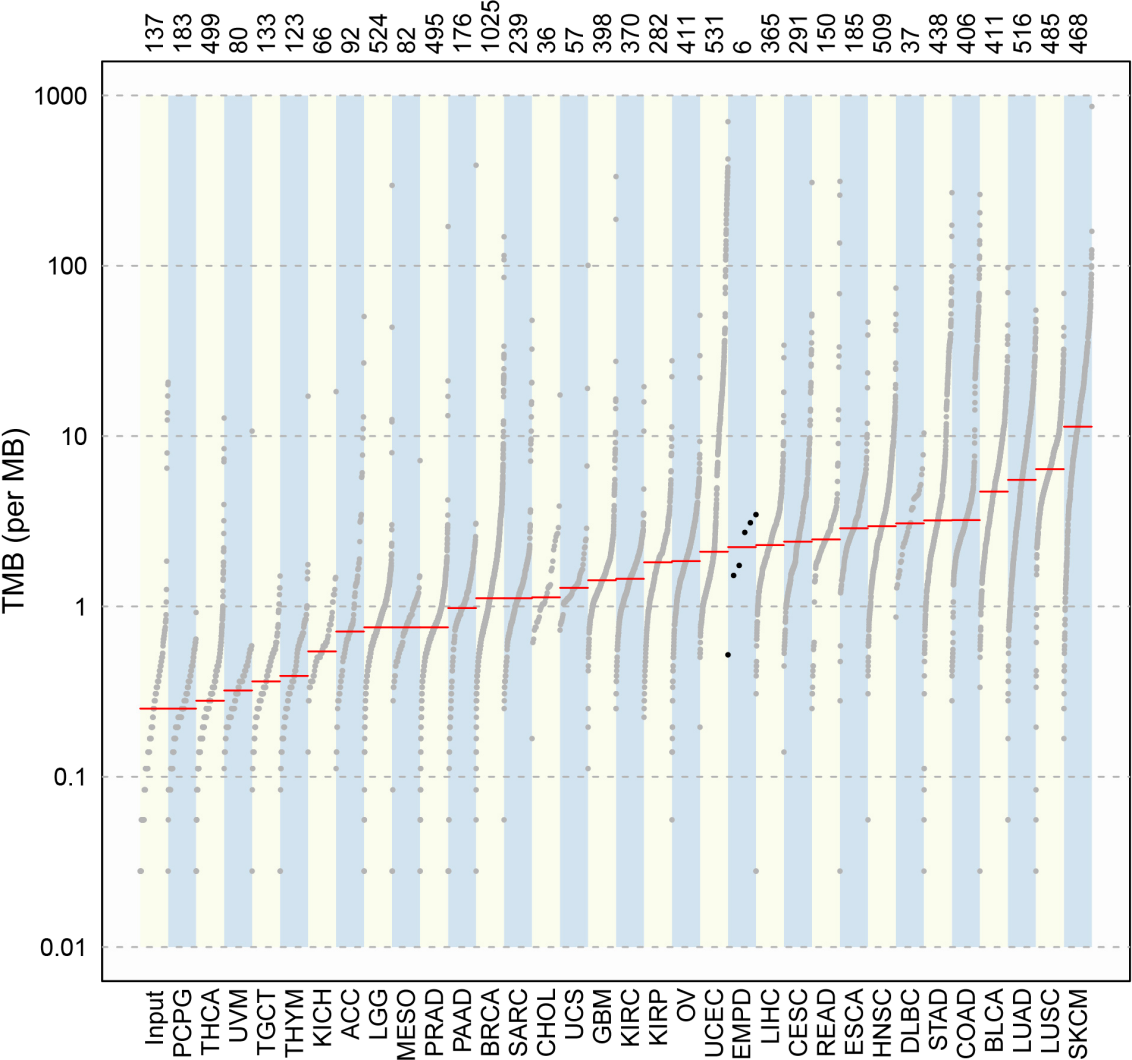
Comparison of TMB between pEMPD and the somatic mutations across 33 human cancer types in TCGA. We can compare TMB in different cancer types and the alignment of the mutation data in a simple and intuitive way.

Compared with the mutation load of 33 cancer types in TCGA cohorts (**Figure 4**), the TMB alignment of pEMPD was close to ﻿the TMB of cervical squamous cell carcinoma and endocervical adenocarcinoma (CESC), uterine corpus endometrial carcinoma (UCEC), liver hepatocellular carcinoma (LIHC), and rectum adenocarcinoma (READ).

### Interactions of somatic mutations

**Figure 5:**
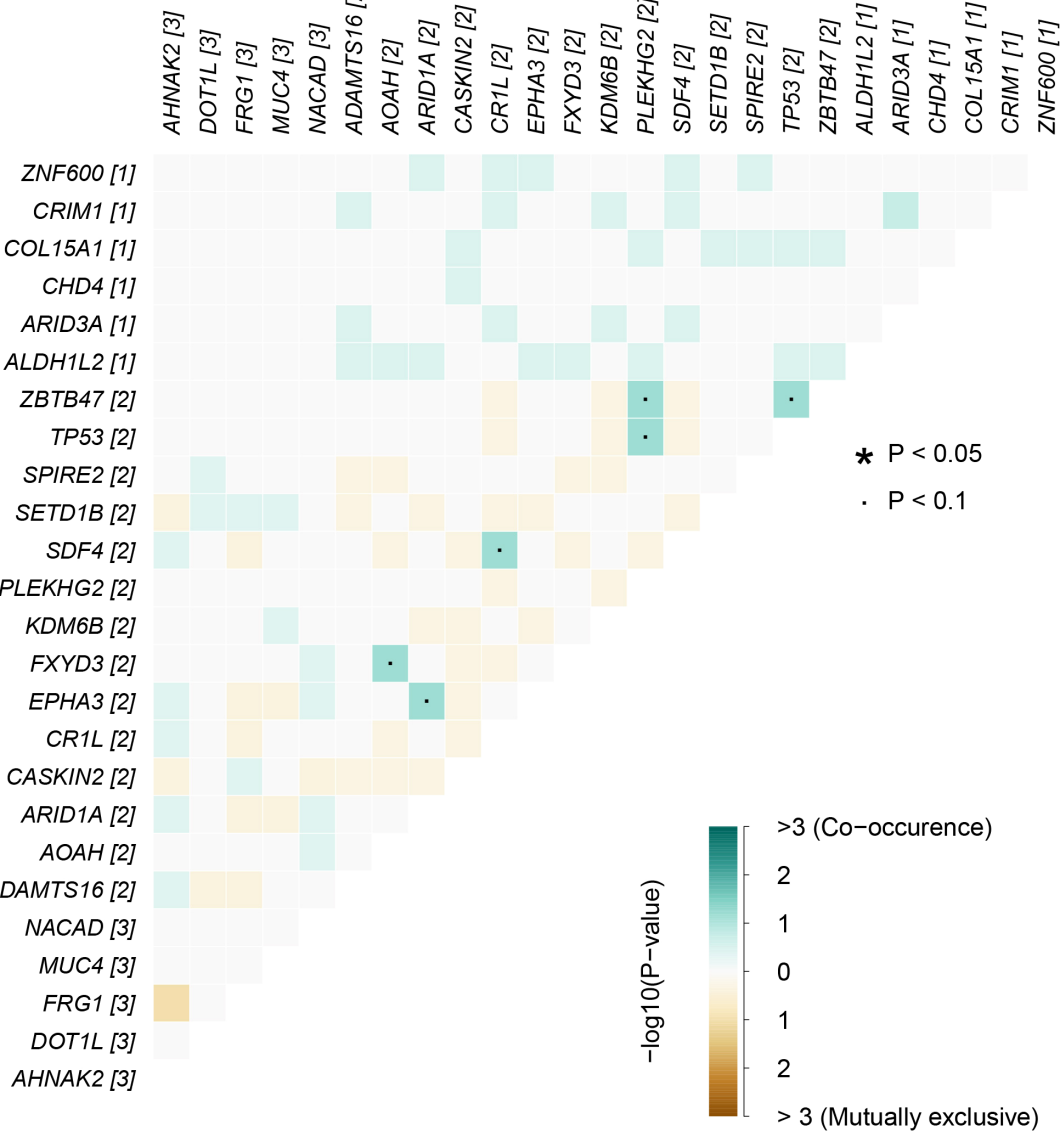
Mutually exclusive or coexisting gene mutations of pEMPD. ZBTB47 and TP53 coexist significantly with PLEKHG2 in the top 20 differential genes. Green is a mutant gene that tends to coexist; yellow tends to be mutually exclusive, and color depth indicates significance. The significant statistical differences were defined as *P <0.05.

In cancers, many genes display coexistence exclusivity in their mutation patterns. In our series, ZBTB47 and TP53 coexisted significantly with PLEKHG2 (**Figure 5**).

## Discussion

Primary EMPD, by definition, is a rare adenocarcinoma originating in the skin (11), which was first described by Croker in 1889. It has a prolonged clinical course with recurrences and eventually distant metastatic spread. The histopathological characteristic for this tumor is PCs, which are atypical large cells with vesicular nuclei and abundant, clear, and, sometimes, eosinophilic cytoplasm (12). Although *in situ* form is most common for EMPD, PCs can invade into dermis and deep structures as invasive EMPD with increasing risk of the lymph node and distant metastasis (13, 14). Controversy exists over pathogenesis for EMPD, including the epidermotropic theory and the transformation theory as we mentioned before. In our research, PCs generally expressed CK7 and CEA and were negative for P63 and CK10, which was consistent with the result of the other previous reports (15–17). As a relatively special biomarker for skin apocrine gland, GCDFP-15 was reported with positive rate up to 30%–90% in EMPD (15, 18). More than half of cases in our study expressed GCDFP-15, which implied that tumor may be related to sweat glands in the epidermis. CK20 is usually negative in primary EMPD, so it can be used to discern the primary from the secondary EMPD, such as originating from colorectal cancer; in our cohort, about 5% of cases expressed variable CK20, which suggested that, in practice, we should use a panel marker including CK20 and other biomarkers to make differential diagnosis. Some previous studies observed that MUC5AC expression tended to be significantly higher in invasive lesions and metastatic lymph nodes than that in *in situ* lesions, which could be used as a marker for identifying high-risk EMPD (19–21), However, we found that MUC5AC lost in invasive pEMPD, which suggested that MUC5AC played a protective role; the decreases may promote tumor malignant potential. This deduction was consistent with the research by Yoshii et al. (22). These converse conclusions may be explained by the different anatomic region as we focused on the samples from penoscrotum. AR, a hormonal receptor, was frequently overexpressed in EMPD( (23, 24). AR protein expression has also been shown to correlate with the invasiveness in EMPD (3), just as our study has shown. On the basis of this, anti-androgen target therapy seemed to be an effective therapy especially for invasive EMPD (25).

In general, our immunohistochemical findings suggested that *in situ* and invasive pEMPD had a similar immunotype, which suggested that the tumor cell might come from intraepdermis sweat gland. Additional studies are needed to confirm this conclusion.

To our knowledge, our study is the first one to compare genomic changes between invasive pEMPD and its *in situ* counterpart. The paired sample mutations detected in our WES testing cohort were quite different from other previous studies (4, 5). This might be interpreted by distinct enrolled samples between the studies: ethnic or geographical difference, and intertumor and intratumor heterogeneity (26).

Tumor progression is an extremely complicated process involving multi-gene mutation and multi-stage evolution. The WES testing showed that missense mutation was most frequent in our cohort, and *C>T* transition presented majority cases. All samples harbored a significantly higher number of somatic alterations. Tumor TMB was found close to CESC, UCEC, LIHC, and READ, which were lower than that found in cutaneous melanoma and cutaneous squamous cell carcinoma.

Invasive pEMPD displayed variable degrees of genetic difference to *in situ* partner including AHNAK2, DOT1L, FRG1, MUC4, TP53, NACAD, CASKIN2, ARID1A, AOAH, and ADAMTS16. MUC4 belongs to membrane-bound mucins localized on chromosome band 3q29. It is a cancer gene aberrantly produced in a variety of cancers and functionally linked to tumor initiation, metastasis, and interaction of tumor cells with the components of the tumor microenvironment (27). However, it is still ambiguous whether MUC4 can play a promoting or inhibiting effect across the cancer types, depending on the particular cancer and cell context (28). Previous studies reported that MUC4 played a critical role in tumor progression and metastasis such as breast cancer (29), ovarian cancer (30), and pancreatic cancer (31). On rare occasions, it can afford protective effect for HNSCC (32). In our research, comparing invasive to *in situ*, half of the paired cases showed MUC4 mutation, hence we supposed it as a biomarker for cancer progression risk detection and intervention treatment. This conclusion should be testified by more data and experiments in the future.

Many pathways involve EMPD carcinogenesis and development except PI3K–AKT pathway (4). ANAK2H mutation appeared in 50% of the cases (3/6) as two missense mutations and one multi-hit mutation, which is consistent with the result from the cBioPortal analysis (**Supplementary Figure 1A**). ANAK2H mutation in cancer types summary of cBioPortal analysis (**Supplementary Figure 1**); AHNAK2 was first discovered at the time when the function of its sister AHNAK nucleoprotein (AHNAK) was explored in 2004 (33). It is frequently overexpressed as a poor prognostic biomarker in a variety of cancers such as breast cancer, papillary thyroid carcinoma (34), pancreatic ductal carcinoma, and clear cell renal cell carcinoma (35). AHNAK2 performs a tumor regulatory function involving multi-faceted processes. Through the MAPK pathway or the TGF-beta/Smad3 pathway, AHNAK2 promoted lung cancer progression, which involves cell proliferation, migration, invasion, and epithelial–mesenchymal transition (36, 37). As for bladder cancer, AHNAK2 overexpression predicted poor overall survival and tumor malignancy; the knockdown of AHNAK2 significantly weakened the invasive capacities of bladder tumor cells (38). AHNAK2 can also carry out its pro-tumor role *via* activating immune microenvironment. Yanan Cui et al. proposed that Del-AHNAK2^mut^ increased the TMB and NAL level, activated cytotoxic T lymphocyte (CTL) effector functions and interferon-gamma (IFN)-γ signaling, and eventually initiated the therapeutic immune response of NSCLC (39). AHNAK2 knockdown was previously reported to activate the Wnt pathway and correlated with tumor immune cell infiltration thyroid cancer (40).

Some limitations of the present study should be noted. First, this is a single-center and retrospective study, and the sample size is relatively limited. Second, we used the WES testing to characterize the genetic alterations of invasive/*in situ* pEMPDs; however, we could not detect non-coding and structure variants. The absence of molecular biology experimental validation was also one of the limitations of our study. Therefore, for future prospective, multi-center and large cohort studies are warranted to confirm our findings and to search for the molecular invasion basis of pEMPD.

In summary, we postulated that the cell origin of pEMPD might be an epidermal sweat gland based on of its immunophenotype. By means of comparing genomic features of invasive/*in situ* pEMPD, we sensitively detected mutations in small FFPE tumor sample by the WES testing. MUC4 and AHNAK2 were the most frequent differential genes in invasive disease compared with that in *in situ*; they may play the role of promoting tumor invasion. Our finding may provide a new insight into tumor evolving and propose potential therapeutic targets for development of pEMPD therapies in the future.

## Data availability statement

The datasets presented in this study can be found in online repositories. The names of the repository/repositories and accession number(s) can be found in the article/**Supplementary Material**.

## Ethics statement

The studies involving human participants were reviewed and approved by Human Ethics Committee of the Shanghai Nineth People’s Hospital, Shanghai Jiaotong University School of Medicine (SH9H‐2019‐T181‐2). The patients/participants provided their written informed consent to participate in this study.

## Author contributions

YRa and TJ conceived, designed, and planned the idea. YR contributed to language editing of manuscript. HZ and JZ analyzed the data. TJ and YRe revised the manuscript. All authors contributed to editorial changes in the manuscript. All authors read and approved the final manuscript.

## Funding

Cross disciplinary as Research Projects (JYJC202106) and Biobank Research Projects (YBKB201911).

## Conflict of interest

The authors declare that the research was conducted in the absence of any commercial or financial relationships that could be construed as a potential conflict of interest.

## Publisher’s note

All claims expressed in this article are solely those of the authors and do not necessarily represent those of their affiliated organizations, or those of the publisher, the editors and the reviewers. Any product that may be evaluated in this article, or claim that may be made by its manufacturer, is not guaranteed or endorsed by the publisher.

## Supplementary material

Supplementary Figure 1 cBioPortal shows the mutation frequency of AHNAK2 gene in different cancer types.
