# Supplementary figures and images for: Cell origin and genome profile difference of penoscrotum invasive extramammary Paget disease compared with its in situ counterpart
Source: Front Oncol. 2022 Aug 24;12:972047. doi: 10.3389/fonc.2022.972047 (PMC9451029; doi:10.3389/fonc.2022.972047)

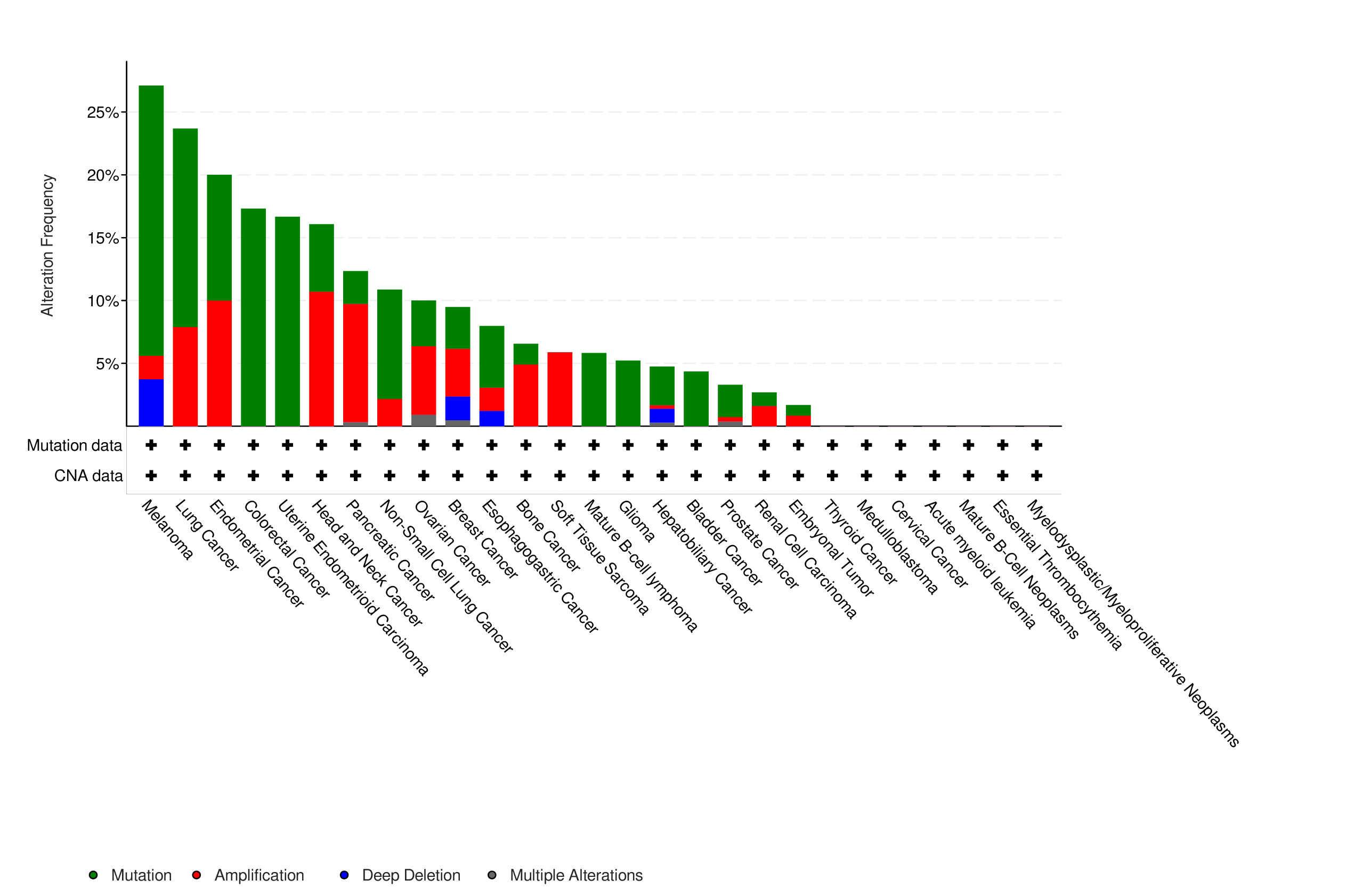

Supplement: Supplementary Figure 1 — cBioPortal shows the mutation frequency of AHNAK2 gene in different cancer types. [file Image_1.jpeg]
